# Exploring health care seeking knowledge, perceptions and practices for childhood diarrhea and pneumonia and their context in a rural Pakistani community

**DOI:** 10.1186/s12913-018-2845-z

**Published:** 2018-01-27

**Authors:** Wafa Aftab, Leah Shipton, Fauziah Rabbani, Kashif Sangrasi, Shagufta Perveen, Aysha Zahidie, Imran Naeem, Shamim Qazi

**Affiliations:** 1 Department of Community Health Sciences, The Aga Khan University, Karachi, Stadium Road, P.O Box 3500, Karachi, 74800 Pakistan; 2 Dalla Lana School of Public Health, University of Toronto, Toronto, Canada; 3 Department of Maternal, Newborn, Child and Adolescent Health, World Health Organization, Geneva, Switzerland

**Keywords:** Developing country, Community health worker, Diarrhea, Pneumonia, Community case management, Child health services, Caregivers, Health care seeking behavior

## Abstract

**Background:**

Where access to facilities for childhood diarrhea and pneumonia is inadequate, community case management (CCM) is an effective way of improving access to care. In Pakistan, utilization of CCM for these diseases through the Lady Health Worker Program remains low. Challenges of access to facilities persist leading to delayed care and poor outcomes. Estimating caregiver knowledge, understanding their perceptions and practices, and recognizing how these are related to care seeking decisions about childhood diarrhea and pneumonia is crucial to bring about coherence between supply and demand-side practices.

**Methods:**

Data was collected from family caregivers to explore their knowledge, perceptions and practices regarding childhood diarrhea and pneumonia. Data from a household survey with 7025 caregivers, seven focus group discussion (FGDs), seven in-depth interviews (IDIs), and 20 detailed narrative interviews are used to explore caregiver knowledge, perceptions and practices.

**Results:**

Household survey shows that most family caregivers recognize main signs and symptoms of diarrhea such as loose stools (76%). Fewer recognize signs and symptoms of pneumonia such as breathing problems (21%). Few caregivers (18%) have confidence in lady health workers’ (LHWs) ability to treat childhood diarrhea and pneumonia. Care seeking from LHWs remains negligible (< 1%). Caregivers overwhelmingly prefer to seek care from doctors (97%). Seventy-five percent caregivers sought care from private providers and 45% from public providers.

FGDs, IDIs, and narrative interviews show that care mostly begins with home remedies and sometimes self-prescribed medicines. Treatment delays occur because of caregiver inability to recognize disease, use of home remedies, financial constraints, and low utilization of community based LHW services. Caregivers do not seek care from LHWs because of lack of trust and LHWs’ inability to provide medicines. If finances allow, private doctors, who caregivers perceive as more responsive, are preferred over public sector doctors. Financial resources, availability of time, support for household chores by family and community determine whether, when, and from whom caregivers seek care.

**Conclusions:**

Many children do not receive recommended diarrhea and pneumonia treatment on time. Taking into consideration caregiver concerns, adequate supply of medicines to LHWs, improved facility level care could improve care seeking practices and child health outcomes.

**Trial registration:**

The trial is registered with ‘Australian New Zealand Clinical Trials Registry’. Registration Number: ACTRN12613001261707. Registered 18 November 2013.

## Background

Early and appropriate treatment is the key to saving lives of children sick with diarrhea and pneumonia. Existing effective interventions delivered at high coverage can prevent majority of deaths due to diarrhea and pneumonia among children under five years of age [1]. However, global coverage for most interventions is below 50% [2]. Facility based care is not adequately accessible in most high mortality areas, especially within the crucial first 24 h after disease onset [3]. Where access to facility-based care is low, community case management (CCM) of diarrhea and pneumonia is one of the most effective interventions for reducing under-five mortality due to these diseases [1].

Apart from accessible care, caregivers’ recognition of disease, timely decision making and care seeking from a skilled health provider also determine disease outcome. Poor family caregiver disease recognition and delayed care seeking from skilled providers leads to preventable morbidity and mortality from diarrhea and pneumonia [4].

A crucial aspect of family caregiver decision making is the choice of health provider. As facility-based care remains inaccessible to the most vulnerable children, many countries have initiated community health worker (CHW) programs to improve access. In Pakistan, lady health workers (LHWs) have been providing CCM for diarrhea and pneumonia to children under five since the inception of the National Program for Family Planning and Primary Health Care (also called Lady Health Worker Program) in 1994. LHWs provide curative services for childhood diseases and dispense medicines (such as zinc and ORS for diarrhea and antibiotics for pneumonia) according to LHWP guidelines. Severe cases are referred to health facilities.

Unfortunately, utilization of community-based health worker services for these childhood illnesses remains low in many low- and lower-middle income countries (LMICs). A systematic review of care seeking practices for childhood illnesses in LMICs, including Pakistan, found that only 4.2% caregivers seek care from CHWs for pneumonia and 5.4% for diarrhea [4].

In Pakistan, where merely 38% children with diarrhea receive oral rehydration solution (ORS) and only 41.5% children with pneumonia receive antibiotics [5], only about 0.7% caregivers seek care from LHWs for suspected pneumonia [6]. Instead, as in other South Asian countries, caregivers in Pakistan prefer to seek care from physicians and most often from the private sector [4]. A number of studies have confirmed these care seeking patterns in Pakistan that are inordinately skewed towards private sector physicians as opposed to public sector facilities or LHWs. However, no studies have explored the reasons for low utilization of LHWs for childhood diarrhea and pneumonia in Pakistan. LHW services has been advocated as a way of improving access to trained health providers for childhood diarrhea and pneumonia but low utilization remains a problem. Therefore, we aim to explore patterns of utilization of LHW services and also to put them in the broader context of how caregivers make care seeking decisions and the considerations that influence these decisions.

This data for this study was collected as part of a randomized controlled trial, Nigraan (Urdu word meaning supervisor), in district Badin in southern Pakistan. The trial tested the effect of a supportive supervision intervention on LHWs’ CCM of childhood pneumonia and diarrhea [7]. We also investigated how caregivers decide whether to seek care from LHWs and the factors that influenced such decisions. Other sources of care including public and private providers were also explored.

The triangulation of quantitative methods with various qualitative methods, including innovative use of narrative medicine interview technique, is meant to provide an insight into care seeking practices and the contextual circumstances in which care seeking decisions are made.

## Methods

### Study design and setting

Nigraan, a cluster randomized trial, was conducted in district Badin in southern Pakistan [7]. The district has an area of 6726 sq. km and a population of about 1.35 million, of which 84% is rural [8]. Badin’s economy is mainly dependent on agriculture and fishing. The literacy ratio of the district is 24.63% percent. Male literacy is three times higher than female literacy [9].

In Badin, primary health facilities include 11 rural health centers and 34 basic health units. Secondary level facilities include one district headquarter hospital and four taluka headquarter hospitals [10]. At community level, a network of about 1100 LHWs, supervised by 36 lady health supervisors (LHSs), caters to maternal and child health needs. A vast network of private care providers also exists in the district.

In this paper, we present triangulated data of caregivers’ knowledge, practices and perceptions about seeking care for diarrhea and pneumonia in children under five from: i) household survey; ii) focus group discussions (FGDs) and in-depth interviews (IDIs); and iii) narrative interviews.

### Quantitative data – Family caregiver household survey

A household survey was conducted with family caregivers of children below five years of age in the catchment area of all study LHSs and LHWs using multi-stage cluster sampling.

A structured questionnaire was adapted from the validated Pakistan Demographic and Health Survey (PDHS) 2012–13 questionnaire. The questionnaire included questions on caregiver knowledge about signs and symptoms of diarrhea and pneumonia, prevalence of signs and symptoms consistent with diarrhea and pneumonia in the last 2 weeks, caregiving practices for the most recent episode of diarrhea and pneumonia, and caregiver perceptions and utilization practices for various categories of care providers. An additional module regarding perceptions and practices about the Lady Health Worker Program (LHWP) was included in the questionnaire. Quantitative data was analyzed using STATA 13. Descriptive statistics from the analysis are presented here.

### Qualitative data – Focus group discussions, in-depth interviews, and narrative interviews

#### Family caregiver focus group discussions and in-depth interviews

FGDs and IDIs were conducted with caregivers of children under-five living in the study area. Respondents were selected through purposive sampling by a social mobilizer utilizing community networks and ensuring representation from all study areas. Seven FGDs and seven IDIs were conducted. One of the authors who has formal training and experience in qualitative research, was present in all FGDs and IDIs assisted by an experienced moderator.

Discussion guides with open-ended questions were used in all FGDs and IDIs. Questions were asked about caregivers’ knowledge, practices and health seeking behavior for diarrhea and pneumonia in children under five, their perceptions about LHWP and the role of LHWs in managing a child with pneumonia or diarrhea in the community. Each FGD had 6–7 participants and lasted for around 60–70 min. Each IDI lasted for around 40–45 min. All FGDs and IDIs were audio-recorded and subsequently transcribed. Manual content analysis was done by one of the authors. All transcripts were read through and annotated where relevant information was found. Various types of information emerging from the annotation was enlisted. Each item in the list was categorized and described. Several common themes emerged from the categorization. At the end, all transcripts were reviewed to make sure that all the necessary information had been captured and appropriately listed, categorized, and interpreted.

#### Family caregiver narrative interviews

Narrative medicine practices are primarily utilized in clinical practice to understand patients’ perspective and elucidate the meaning underlying their situations [11]. In this study, we use narrative medicine to frame interviews with caregivers of children under five to understand the viewpoints that underlie care seeking decisions for pneumonia and diarrhea. We conducted narrative interviews with multiple caregivers in each household to inform a holistic understanding of care seeking practices and decision making.

Caregivers of children under five were chosen by purposive sampling based on their caregiver relationship (e.g. father, mother, or grandparent), socioeconomic status, and urban or rural setting. We interviewed at least two participants from each household who were differently placed in their ability to influence decision making, for example: a mother and a grandmother; or a father and a mother. Each respondent was interviewed separately and privately. Twenty caregivers from 11 households were interviewed.

The interview tool was developed with support from an international narrative medicine expert. Three of the authors received training in conducting and analyzing narrative medicine interviews. All three authors were present in each interview. The tool asked questions that explored participants’ perceptions of healthy and sick children, their health seeking behavior, interactions with the health system, and management of treatment plans. Interviews lasted between 45 to 105 min.

Interviews were audio recorded and transcribed. The data was analyzed by manual content analysis. Thematic analysis was conducted on interview transcripts and evolving themes and codes were compared among all interviews. Each transcript was read and analyzed by two researchers. Emergent codes, themes, and interpretations were later compared by the two researchers. Descriptive accounts of interviews were created as concise ‘stories’ highlighting contextual details of important themes.

### Triangulation of qualitative and quantitative findings

This study used the convergence triangulation model by Creswell et al. 2011, wherein researchers collect and analyze both quantitative and qualitative data during the same phase of research and then merge the results into an overall interpretation [12]. In accordance with the model, data from the three methodologies was collected and analyzed independently and then their findings were compared, contrasted and interpreted. Once separate data analyses were done, three authors reviewed data from all sources together to create common themes, which illustrated caregiving practices and their context. Relevant data from the three sources was organized under five themes. Authors discussed and debated all disagreements to develop themes that appropriately reflected participant data and viewpoints regarding care seeking for their children under five with pneumonia and diarrhea. After preliminary data categorization, qualitative transcripts and quantitative data were reviewed to ensure that all relevant information had been captured.

### Ethics, consent and permissions

Ethical approval was taken from Aga Khan University’s Ethics Review Committee (2650 – CHS – ERC – 2013) and World Health Organization Ethics Review Committee (MCA00113). Written informed consent was obtained from all participants.

## Results

Findings from the three methodologies contributed insights to the caregiving experience in Badin. Among the surveyed family caregivers, 99% were women, 76% were between the ages of 25 and 45 years, and 97% were married. Caregivers were evenly divided in five wealth quintiles, calculated using DHS methodology. Most caregivers (73%) had no or less than 1 year of formal education. Forty-five caregivers participated in FGDs and IDIs. All participants were women between the ages of 18–50 with at least one child under five. Most participants had no formal education. The 20 narrative interview participants included 13 women and seven men. In terms of caregiver relationship, we interviewed seven mothers, six fathers, three grandmothers and four other extended family members. In contrast to narrative interviews, almost all family caregivers in the survey, FGDs and IDIs were mothers of children under five.

The triangulated findings of the study are described under the themes of: i) disease burden; ii) caregiver knowledge; iii) home management; iv) community based management; and v) facility-based management. Within each theme, the caregiver household survey contributes quantitative data; the FGDs and IDIs illustrate broader community opinions; and narratives present deeper insight into the story behind caregiver decisions. Table 1 provides a pictorial representation of various modes of caregiver recognition and response to childhood diarrhea and pneumonia utilizing a framework by Colvin et al. [13]

**Table 1:** Modes of caregiver recognition and response to childhood diarrhea and pneumonia in Badin. *Adapted from Colvin* et al. [13]

Caregiver recognition and response	Seeking advice and negotiating access	Using the middle layer between home and clinic	Accessing formal bio-medical services
Actors: Caregivers, immediate and extended family members.	Actors: Caregivers, extended family, community members.	Actors: Caregivers, lady health workers, medicine sellers.	Actors: Caregivers, extended family, community, private sector providers, public sector providers, employers.
Factors affecting response: Knowledge of signs and symptoms of diarrhea, dehydration, and pneumonia; previous experience with child illnesses in household or community.	Factors affecting response: Perception of disease severity, belief and experience with home remedies, influence of household elders.	Factors affecting response: Experience with self-medication, knowledge of LHW ability to treat childhood disease, belief in LHWs’ ability to provide appropriate care, availability of medicines with LHWs.	Factors affecting response: Financial consideration, availability of transport, disease severity, location of health facility, perception of public vs private provider competence, attitude, and attentiveness; caregiver work replacement.

### Disease burden

From the baseline survey, the prevalence of pneumonia in children under five in the two-week period before the survey was 19.5% (*N* = 6969) and the prevalence of diarrhea was 18.5% (*n* = 6969). According to FGDs and IDIs conducted with caregivers, diarrhea, vomiting and fever are viewed as the most common childhood illnesses. These comments are echoed by the narratives which reveal that caregivers are overwhelmed by the needs of their sick children and the disruption it causes to their daily lives. In particular, caregivers discussed the inconvenience caused by diarrhea but were less descriptive of the ways pneumonia impacts their life.

In the narrative story of a father of three children under five years of age living in a rural area of Badin, it was revealed that he and his wife work as laborers, but struggle to meet the financial needs of their family. While telling his story of care giving, he was aware of the seriousness of diarrhea in his community. Despite hardships he feels empowered and confident in his ability to seek appropriate treatment when his children become sick.

*“ … (Diarrhea) happens to my children too. Diarrhea is most common, but our children also get sick from pneumonia. Previously we had open latrines so the virus spread to the other people of our village, but now we have almost resolved this problem. The (elder children) know (how to take care of younger children when they are sick) because here the ratio of healthy children is very less. (Children) also get sick (because) we have poverty here and the health center is far away from the village. We try to keep our child safe from these diseases because of some incidences (that) happened in which some children died because of diarrhea and pneumonia…* ”*– Family caregiver narrative interview*

### Caregiver knowledge

When asked about the symptoms of diarrhea in the survey, majority of participants reported loose motions (76%), weakness (33%), dehydration (12%), fever (10%) and abdominal pain (7%). For pneumonia, the bulk of respondents reported fever (71%), cough (56%), chest congestion (22%) and breathing problems (21%) as the main symptoms.

Most caregivers participating in FGDs and IDIs used the same symptoms and signs to correctly describe pneumonia (fever, cough, shortness of breath, chest congestion and chest in-drawing) and diarrhea (frequent loose stools, weakness, and lethargy). For example, one caregiver explained how she checks for dehydration when her child has diarrhea:

*“We can also get idea by checking the skin of the child. Water is coming out of the body so skin will become dry and child will demand for more and more water” - Family caregiver FGD*

While discussing the causes of pneumonia and diarrhea, caregivers attributed these diseases to inadequate personal hygiene, poor environmental sanitation and to a lesser extent bottle feeding, poor maternal nutrition, and weather changes. One caregiver stressed the importance of personal hygiene as a preventative measure:

*“Children should not be allowed to play in mud, their clothes must be clean, and they should take bath twice a day” - Family caregiver IDI*

In another discussion, a caregiver focused on how unhygienic food causes diarrhea:

*“We should avoid outside food they are unhygienic and flies are sitting on those foods so when child eats those foods (s/he) gets diarrhea and other illnesses” - Family caregiver FGD*

Caregiver narratives revealed more in-depth knowledge of diarrhea symptoms and causes. While describing their caregiving experience, caregivers listed loose stools, sunken eyes, weakness, vomiting, dry skin, fever, and loss of appetite as signs and symptoms of diarrhea. They also gauged the severity of diarrhea by observing the frequency with which their child passed stools. Caregivers also narrated with frustration their attempts to protect their children from the numerous causes of diarrhea. The causes mentioned were: poor water quality and sanitation infrastructure, unhygienic and spicy food, hot weather, poor personal hygiene, and exposure to dust and garbage. Conversely, most caregivers hesitated when listing signs and symptoms specific to pneumonia and none mentioned its direct causes. Rather, the few caregivers that discussed causes of pneumonia attributed it to unhealthy food, (e.g., chips and ice cream), cold weather, and contaminated water.

Caregivers credited their own experiences caring for children as ways that they learned the signs and symptoms of pneumonia and diarrhea. Eighty percent caregivers in the survey credited their awareness of signs and symptoms of diarrhea and pneumonia to their own knowledge. Doctors, family members, and LHWs were identified as sources of knowledge only by 7%, 9%, and 0.3% caregivers, respectively.

The narratives with experienced vs. inexperienced caregivers shed further light on how caregivers come to claim knowledge about disease signs, symptoms, and causes as their ‘own’. All caregivers discussed learning from elders and family members at some point in their caregiving experiences. Additionally, some caregivers mentioned advice from doctors and non-governmental organizations working in the area. Inexperienced caregivers access knowledge from these sources when their children get sick. Contrastingly, experienced caregivers can respond to an episode of illness using their ‘own’ knowledge without advice from others. However, experienced caregivers’ ‘own’ knowledge is the accumulation of advice from these same sources over many care giving episodes through years of care giving experience. One caregiver illustrates this summation of knowledge:

*“I have been taking care of my niece and nephew so I came to know [about pneumonia and diarrhea] by my experiences. I was also caring for my own children and doctor also tell us sometimes.” - Family caregiver narrative interview*

### Home management

Most caregivers’ initial response to their child being sick is to manage it at home by changing their diet, using various home remedies, and/or self-prescribing medicines. While managing diarrhea at home, only about one-third (33%) of caregivers in the survey mentioned increasing their child’s fluid intake. A similar proportion (35%), opt to decrease the amount of fluids given, while the remaining one third maintained their child’s usual fluid intake. On the other hand, the majority (62%) of caregivers lessen their child’s food intake during an episode of diarrhea. The remaining caregivers maintain their child’s usual food intake (34%) or increase it (4%). The survey showed that caregivers follow similar feeding practices for pneumonia.

In FGDs and IDIs, caregivers explained why they followed these feeding practices. For example, one caregiver explained why she increases her child’s food intake:

*“We increase the amount of feed because in diarrhea the water is coming out from body so the child needs more”. - Family caregiver FGD*

While another caregiver supported giving lesser food to a sick child by saying:

*“We give them less food, already the child is sick.” - Family caregiver FGD*

In FGDs and IDIs most caregivers said that they opt for a soft diet of rice, yogurt, and bananas along with ORS when their children have diarrhea. For pneumonia, caregivers continue breast feeding and give warm foods such as soup, warm milk, egg, and honey while avoiding cold water and oily and sour foods.

Caregivers also utilize home remedies to manage their child’s diarrhea (47%) and pneumonia (28%). The most common home remedies reported for diarrhea are yoghurt, banana, rice, cardamom and salt sugar water. The most commonly used remedies for pneumonia are herbal tea (joshanda and kahwa), cardamom, honey and anise. Most caregivers in FGDs and IDIs mentioned using home remedies for mild cases of diarrhea and pneumonia before seeking care from a health provider.

*“We try to treat them at home. We give flagyl (metronidazole), ORS (for diarrhea), despite that if the child’s condition is not getting better then we take the child to hospital.” - Family caregiver IDI*

In the narratives, caregivers detailed how knowledge about home remedies is primarily passed down from elders in the household and community to younger caregivers. Inexpensive and readily available home remedies are a routine first response to manage child sickness, particularly if the symptoms and signs are mild. Caregivers who prefer using home remedies tend to have a strong faith in God, they trust in or have witnessed the usefulness of these remedies, and they are influenced by the presence of elders in the household. More educated caregivers favor using home remedies relatively sparingly, but if elders in the household prefer using home remedies before seeking care then educated caregivers abide out of respect.

Additional to changes in feeding practices and home remedies, 33% caregivers in the survey report using self-prescribed medicines for pneumonia and 72% use store bought ORS for diarrhea. Many caregivers in FGDs and IDIs added that they use metronidazole syrup purchased from general stores to treat diarrhea. Narrative interviews showed that caregivers opt for self-prescribed medicines prior to health seeking from doctors for convenience and cost-saving. For example, caregivers save prescriptions or leftover medicines from doctors to reuse when their child becomes sick again. This was the situation described a mother of two children under the age of five living in a middle-income household of an urban area:

*“…they fall sick very often, after every 1 to 2 months. So we don’t go to the doctor every time, we use the same medicines that we got from the doctor in the previous visit.”* - *Family caregiver narrative interview*

### Community based management - care seeking from LHWs

The survey shows that family caregivers rarely (< 1%) consult LHWs for diarrhea and pneumonia care in children under five. This dynamic was reiterated by caregivers during narrative interviews who primarily discussed seeking care from doctors. Only when prompted about seeking care from LHWs did caregivers mention them only to say that they do not usually consider going to them. This stark absence of LHWs as care providers from narrative discussions can be explained by the survey data. Only about 18% of caregivers think that LHWs are capable of providing pneumonia and diarrhea care to children. Instead, caregivers predominantly view LHWs as “polio workers” (90% of caregivers report that LHWs provided polio vaccination during their routine visits).

In IDIs and FGDs most caregivers mentioned vaccination, giving polio drops and providing health information as LHWs’ main functions in the community. A few caregivers also mentioned family planning services. Only a few caregivers spontaneously mentioned providing treatment for diarrhea and pneumonia as a routine LHW function. Most caregivers thought that LHWs cannot provide proper care for diarrhea and pneumonia because they do not have medicines. Many were of the opinion that LHWs could provide satisfactory care for diarrhea and pneumonia if they had medicines. For instance, one caregiver talking about LHWs said:

*“LHWs give good suggestions but they don’t get medicine (nowadays) so that they can provide us. If they give medicine then people will get satisfied” - Family caregiver FGD*

Another caregiver talking about care seeking from LHWs said:

*“We ask from lady health worker, if she doesn’t have medicine then we prefer doctor.” - Family caregiver FGD*

### Facility based care seeking

When caregivers perceive their children to be severely sick with diarrhea and pneumonia they may decide to take their child directly to a doctor. Otherwise, caregivers manage their child’s sickness at home and decide to seek care if symptoms and signs persist or worsen. In household survey, caregivers overwhelmingly preferred (97%) doctors as their first point of contact with the health care system.

While deciding on a health care provider, key decision makers in the household consider many factors. These were described in detail in the narrative interviews. In joint family systems, these decision makers are mostly the mother, the father, and the paternal grandmother of the child. However, it is often the grandmother who has the most consistent influence. In all households, the grandmother at the least has her opinion considered while in some households she is the sole decision maker. For example, even if the mother and father prefer a different health seeking choice, they would defer to the grandmother’s opinion out of respect.

Numerous financial and practical considerations affect care seeking decisions. If decision makers have access to sufficient shared family savings they can act on their decision more promptly. Otherwise, they have to borrow money from neighbors, family members, friends, or employers. In most cases it is the father who is responsible for leveraging these social contacts for financial support.

Simultaneously, decision makers figure out other practical factors involved in accessing care. First, if the mother cannot take the child to the doctor by herself and her husband is not home, then accompaniment is required from her mother-in-law or a male relative. Unless the doctor is within reasonable walking distance, the household has to arrange transport that is affordable and available. Transportation difficulties delay care seeking, particularly in rural areas. For example, if a child falls sick at night caregivers will wait until morning to seek care when transportation is more easily available.

Caregivers who are taking their child to the doctor must also consider who will cover their household or work responsibilities in the meanwhile. For example, caregivers working outside the home may have to find someone, often a family member, to replace them otherwise they have to forego their wages for the day. Within the household, female relatives organize the distribution of responsibilities (e.g. cooking, cleaning, child care.) if the mother has to go.

These factors take time to address resulting in delay in care seeking. In the baseline survey, about 84% and 88% of caregivers mentioned seeking care for diarrhea and pneumonia respectively within 48 h of onset of illness. In FGDs, caregivers discussed how the various factors mentioned above influence the timing of care seeking:

*“We observe for whole day and night, if the child condition gets worst then we rush to the doctor” - Family caregiver FGD*

*“(We seek care) on same day, if child gets sick in morning and doesn’t get better till evening.” - Family caregiver IDI*

Caregivers also explained how household financial situation impacts the timing of care seeking:

*“If we cannot afford then we take the child to the doctor after 8–10 days” - Family caregiver FGD*

According to the survey, among caregivers who sought care from a doctor, 75% reported seeking care from private sector while 44% chose government sector. These categories were not mutually exclusive and some caregivers sought care from both sources.

Narrative interviews further elaborate on these health seeking choices. Caregivers acknowledge that government facilities have free consultation and medicines, but associate government facilities with long waiting times, staff shortages, inattentive behavior of staff, and poor quality medicines. On the other hand, while private facilities charge fee for consultation and mostly just give prescriptions for medicines, they have shorter wait times, and private doctors are considered more attentive. Caregivers expect a ‘good’ doctor to examine and diagnose their child properly, address their concerns attentively, give or prescribe good quality medicines, and treat their child successfully on the first visit. Caregivers perceive private doctors to possess more of these qualities as compared to government doctors. Thus they prefer seeking care from private doctors. However, in practice, caregivers seek care from private doctors only if they have the financial means. If they do not have the financial means, caregivers tend to seek care from government doctors initially, and shift to private care if their child’s sickness does not abate. This sequence of care seeking was explained by one caregiver in an FGD as follows:

*“First we take medicine from government doctor if that medicine doesn’t work then we rush to the private doctor.” – Family caregiver FGD*

## Discussion

Our results show that most family caregivers utilize knowledge accumulated through family and personal experiences as the main source of information about pneumonia and diarrhea care. Almost invariably the care process begins with home remedies sometimes accompanied or followed by self-prescription of medicines. Interestingly, even in the presence of a large community health program, care seeking from LHWs for childhood pneumonia and diarrhea remains negligible. Instead, caregivers overwhelmingly prefer to seek care from doctors. If finances allow, private doctors, who caregivers perceive as more responsive, are preferred over public sector doctors. Decision making about care seeking involves the whole family, and sometimes the larger community, rather than just the child’s parents. Financial resources, availability of time, support for household chores by family and community determine whether, when and from whom caregivers seek care.

While caregivers in Badin are aware of causative factors of diarrhea, pneumonia etiology is not well understood. Previous studies from Pakistan and other developing countries show that caregivers associate pneumonia with non-specific factors such as cold weather but not germs [14–16]. Similar beliefs about pneumonia have been documented in a study in Pakistan where participants did not consider pneumonia to be contagious [16]. Inability to recognize the contagious nature of pneumonia could prevent caregivers from adopting measures that might prevent spread within household and in community. Improved caregiver knowledge about preventive factors such as immunization, exclusive breastfeeding, adequate nutrition, good hygiene, and reduction in indoor air pollution could decrease pneumonia incidence in children in Badin.

Caregivers recognize the role of environmental sanitation in preventing diarrhea and are frustrated by the lack of proper sanitation in their communities. Clearly such systemic efforts are beyond the remit of individual caregivers. However, the substantial capacity of such environmental interventions to sustainably reduce diarrhea incidence, along with reduction in related health and resource costs, necessitates more focus on them.

Caregivers in Badin are aware of the most important signs and symptoms of childhood diarrhea but less so of pneumonia. More specific signs of diseases such as signs of dehydration for diarrhea and chest in-drawing/fast breathing for pneumonia are not well known. Studies from Bangladesh [14] and India [15] also demonstrate that most caregivers are unable to recognize specific signs and symptoms of pneumonia. Health education of caregivers and other community members for better recognition of signs and symptoms of pneumonia is vital for early disease recognition and care [14, 17]. LHWP is well placed to play this role if it can provide its services effectively.

Caregivers in Badin commonly use home remedies. While home remedies are seen as cheap and readily accessible, their use has been associated with delayed care seeking as caregivers wait for the remedy to take effect before seeking care [18, 19]. As many caregivers cannot recognize signs and symptoms of disease, time spent waiting for home remedies to work may contribute to poor health outcomes among children with severe illness. This reinforces the need for early assessment by a skilled provider who can diagnose and assess disease severity. Home remedies may be used exclusively or alongside medications in consultation with the provider.

When it comes to seeking care, caregivers in Badin clearly prefer to go to doctors; very few choose to seek the services of LHWs. This is despite the fact that LHWs are easily accessible in the community and provide home consultation and medicines (if available) for free. The reasons for low utilization of community based provider services in our study population may have to do with demand- as well supply-side factors. Few caregivers in Badin trust the ability of LHWs to provide appropriate care for diarrhea and pneumonia. A study from Uganda shows that trust in CHWs and level of community awareness leads to higher utilization of CHW services [20]. In Badin, LHWs’ prestige in the community suffers due to lack of commodities and medicines [21, 22]. CHWs’ respect and prestige is related to both CHWs’ own motivation and with community’s utilization of her services [14, 15, 22]. Ensuring that LHWs have medicines will not only improve utilization for diarrhea and pneumonia but also allow her to perform her role with more motivation and effectiveness.

The fact that financial resources often determine the timing and kind of care sought has equity implications. A well performing community health program can significantly mitigate the effects of these financial barriers [23]. However, currently the LHW program is not adequately performing that function. Improved LHW services, with higher community utilization and better quality of services at referral health facilities could remove that barrier and improve health outcomes for disadvantaged communities.

The predilection for doctors may also be due to the large presence of private doctors in the community who are at times more easily accessible than public sector facilities. Our finding that caregivers prefer private health providers over public providers and that they see private providers as more competent and responsive is in keeping with research done in Pakistan and other countries in South Asia [4].

One potential limitation of this study is the usual challenge of converging quantitative and qualitative data to arrive at meaningful results. To prevent methodological differences from creating a bias and maintain transparency we have ensured clearly pointing out data sources for all data quoted in the results. Secondly, most of our in-depth analysis of decision making practices comes from narrative stories with a limited sample size. Even though we made efforts to have a balanced sample in terms of gender and socio-economic status, it is possible that this data may not be completely representative of broader trends in Pakistani rural population.

## Conclusions

This study shows that caregivers of under-five children in Badin have to make many choices in seeking care for illness. These choices are shaped by a number of social, financial, and health system factors. Health service delivery, from the community to the health facility, must take these concerns into consideration to facilitate correct care seeking decisions by caregivers. In the short term, ensuring timely, appropriate care seeking for children sick with diarrhea and pneumonia means removing the knowledge and disease recognition deficiencies among caregivers, improving LHWs’ capacity to provide quality care, enhancing facility based health infrastructure, better transport options, and removing financial barriers to care seeking.

Reducing childhood diarrhea and pneumonia related morbidity and mortality will require rigorous efforts to scale up evidence-based interventions, especially community-based preventive, promotive and therapeutic strategies, as well as availability of commodities and health workers in primary care. A lack of appropriate care seeking for ill children by community care providers is a major challenge requiring both demand creation as well as provision of services. In the long term, broader socioeconomic and cultural determinants of care seeking should be addressed in order to influence care seeking practices.

## Abbreviations

CCM: Community Case Management
CHW: Community Health Worker
FGD: Focus Group Discussion
IDI: In-depth Interview
LHS: Lady Health Supervisor
LHW: Lady Health Worker
LHWP: Lady Health Worker Program
ORS: Oral Rehydration Solution
WHO: World Health Organization
